# Current status and future perspectives of clinical practice for gaming disorder among adolescents in Japan: A preliminary survey in Sapporo

**DOI:** 10.1002/pcn5.4

**Published:** 2022-03-04

**Authors:** Masaru Tateno, Ayumi Takano, Takanobu Matsuzaki, Susumu Higuchi

**Affiliations:** ^1^ Tokiwa Child Developmental Center Tokiwa Hospital Sapporo Hokkaido Japan; ^2^ Department of Mental Health and Psychiatric Nursing Tokyo Medical and Dental University Bunkyo‐ku Tokyo Japan; ^3^ Department of Psychiatry National Hospital Organization Kurihama Medical and Addiction Center Yokosuka Kanagawa Japan

## AIM

Digital games are the most common leisure activities for adolescent males in Japan. The national survey on gaming (*n* = 5,096, 10–29 years, 2546 males) demonstrated that 85.0% of the total respondents had played digital games in the past 12 months.^1^ Another nationwide survey revealed that the rate of internet use for gaming was 83.7% in 10‐year‐olds (*n* = 288) and 81.4% in 16‐year‐olds (*n* = 404).^2^ As a result of the popularity of online gaming, excessive gaming has become a major social problem among adolescents.^3^

Because the 11th edition of the International Classification of Diseases (ICD‐11) includes gaming disorder (GD) as a psychiatric disorder,^4^ it is expected that more people will pay attention to problems related to excessive gaming. We conducted a preliminary survey of clinicians to understand the current state of clinical practice for GD and to investigate what kind of difficulties they are experiencing.

## METHODS

The subjects of this study were child and adolescent psychiatrists, general psychiatrists, and pediatricians who were engaged in the mental healthcare of adolescents in Sapporo, Japan. With regard to the locus of this study, Sapporo is in northern Japan and its population is approximately 1.95 million. Since child and adolescent psychiatry is not a separate specialty in Japan, the clinician certified by the Japanese Society for Child and Adolescent Psychiatry (JSCAP) is regarded as a child psychiatrist.^5^ In this study, based on their self‐reporting, clinicians practicing in child psychiatry clinics were categorized as Department of Child Psychiatry in the Table 1 regardless of their certification status. General psychiatrists and pediatricians who practice at clinics/hospitals registered as cooperating medical institutions in the Sapporo Child Mental Health Concierge, which is a public telephone consultation service for children's mental health concerns, were invited to participate in this survey. Initially a questionnaire was mailed to all clinicians working with the mental health problems of adolescents in this area. Study subjects were requested to answer the questionnaire anonymously, and their age group and years of clinical experience were requested by checking boxes.

**TABLE 1:** Summary of the socio‐demographics of the respondents and their answers

Department^a^	Child psychiatry	22 (66.7%)
	General psychiatry	12 (36.4%)
	Pediatrics	7 (21.2%)
Place of work	Clinic	18 (54.5%)
	General hospital	8 (24.2%)
	Private hospital	6 (18.2%)
	Others	1 (3.0%)
Clinical experience (year)	<10	4 (12.1%)
	10–19	7 (21.2%)
	≥20	21 (63.6%)
	Blank	1 (3.0%)
Number of GD^b^	Median	2.0 (0–70)
Number of GD^b^	Mean	4.3 ± 5.3 (0–70)

*Note*: The results are expressed as the mean ± standard deviation (SD).

Abbreviation: GD, gaming disorder.

^a^ Multiple answers possible.

^b^ The number in the past 12 months.

Since it was assumed that many clinicians would not be familiar with the diagnostic guidelines for GD in ICD‐11, the following explanation was provided at the beginning of the questionnaire: “In the ICD‐11, gaming disorder is defined as a dysfunctional pattern of gaming, characterized by: a) impaired control; b) an increasing priority given to gaming; and c) a continued involvement in gaming despite negative consequences. The maladaptive gaming pattern has to be manifested over an extended period of time (typically 12 months), and cause significant impairment in social functioning.”

The questionnaire asked for the socio‐demographics of the respondents and the number of patients with GD they had treated as defined by ICD‐11 over the last 12 months, followed by questions about the chief complaints of patients with presumptive excessive gaming at the first visit and related therapeutic interventions employed at each institute. The study protocol was approved by the ethics committee of Tokiwa Hospital (TH‐2011‐01).

## RESULTS

Thirty‐three subjects responded to the questionnaire with the socio‐demographics summarized in the Table 1. The results suggested that many clinicians were seeing cases with GD and excessive gaming‐related problems.

When asked why patients with GD came to see them, only 12 (36.4%) of the subjects answered that their patients' chief complaint was excessive gaming. The more common reasons given by patients who were found to have GD were complaints of school refusal/absenteeism 28 (84.8%) and discovery of GD while visiting the clinic regularly for other psychiatric/developmental disorders 16 (48.5%). Additionally, 14 (42.4%) had shown violence and nine (27.3%) had seen financial billing problems arise. None of the respondents answered that they provided specialized treatment to GD.

## DISCUSSION

The prevalence of GD reported so far is highly variable.^6^ Previous studies on the prevalence of GD have used a variety of self‐rating measures: scales developed for conventional offline‐gaming, scales based on uncertain definitions of GD, and scales for internet addiction (multipurpose). More than anything, it is very difficult to distinguish between those who enjoy games moderately as pleasure and those who could be diagnosed as having GD, the issue being one of degree. Our results demonstrated that only five respondents (15.2%) answered that the number of cases they considered to be GD was 0. In other words, presumably clinicians who deal with adolescents' mental health issues are often consulted for gaming‐related problems. However, none of the respondents provided specialized treatment to GD. Thus, we urgently need to develop guidelines to provide a standardized treatment to GD.

In Japan, school refusal/absenteeism and subsequent severe social withdrawal, *hikikomori*, have become major social problems.^7^ According to a national school survey, the rate of school refusal/absenteeism in junior‐high schools was reported to be 3.9%.^8^ Although many adolescents visit medical institutions with a complaint of school refusal/absenteeism, about 85% of the clinicians suggested that such students may also have problems with excessive gaming, and as a result it might be necessary first to deal with excessive gaming in order to prevent prolonged social withdrawal. It is necessary to identify adolescents with possible GD at an earlier stage by using reliable screening tools.^9^

Our results revealed that violence and large bills related to gaming are also common and serious problems. In addition, about half of the respondents indicated that excessive gaming occurred during their follow‐up period. In Japan, it has been reported that many adolescent patients visiting medical institutions regularly had neurodevelopmental disorders (NDD) such as attention deficit hyperactivity disorder and appearance on the autism spectrum.^10^ Because NDD is known to be a risk factor for GD,^11^ it is important to recognize the early signs of excessive gaming and to provide preventive interventions such as lifestyle guidance and familial psychological education from an early stage.

## PATIENT CONSENT STATEMENT

Patient consent statement information is not available.

## CLINICAL TRIAL REGISTRATION

Clinical trial registration information is not available.

## FUNDING STATEMENT

This study was supported by a Health Labor Sciences Research Grant (20GC1022: Research on the establishment of diagnosis and treatment for gaming disorder).

## CONFLICTS OF INTEREST

The authors declare no conflicts of interest.

## ETHICS APPROVAL STATEMENT

The study protocol was approved by the ethics committee of Tokiwa Hospital (TH‐2011‐01).

## AUTHOR CONTRIBUTION

M.T.: Conception and design of the study, acquisition and analysis of data, drafting the manuscript. A.T.: Conception and design of the study, reviewing and editing the manuscript. T.M.: Conception of the study, reviewing and editing the manuscript. S.H.: Conception of the study, reviewing and editing the manuscript.

## Data Availability

The data of this study are available from the corresponding author upon reasonable request.
